# Supplementary material for: Adaptive DDoS detection mode in software-defined SIP-VoIP using transfer learning with boosted meta-learner
Source: PLoS One. 2025 Jun 26;20(6):e0326571. doi: 10.1371/journal.pone.0326571 (PMC12200684; doi:10.1371/journal.pone.0326571)
Supplement: Attachment — Submitted filename: review.docx [file pone.0326571.s001.docx]

Review of the Paper: "Adaptive DDoS Detection Mode in Software-Defined SIP-VoIP Using Transfer Learning with Boosted Meta-Learner"

The paper proposes a transfer learning-based DDoS detection model for SIP-VoIP using BiGRU, BiLSTM, Random Forest, and XGBoost. While the topic is relevant and the approach promising, several areas require major revision to enhance clarity, justification, and practical applicability.

Key Revisions Needed:

- Classifier Justification: Explain why BiGRU, BiLSTM, and Random Forest were chosen over other models. Compare with alternative deep learning classifiers.
- Dataset & Preprocessing: Provide details on feature selection, normalization, and handling of class imbalance. Clarify preprocessing steps.
- Related Work: Expand comparison with existing SIP-VoIP DDoS detection models and highlight novelty.
- Results & Explainability: Discuss false positives, false negatives, and model interpretability. Include confusion matrices, ROC curves, or SHAP analysis to strengthen insights.
- Abstract & Conclusion: Add specific performance metrics and emphasize practical implications. Ensure clear takeaways.
- Practical Deployment: Address real-time implementation, IDS integration, and robustness against adversarial attacks.
- Grammar & Readability: Improve structure, clarify research gaps, and refine writing for better comprehension.

**Recommendation: Major Revision**

The paper presents a strong concept but requires significant improvements in methodology, dataset details, result interpretation, and writing clarity before it can be considered for publication.
