# Supplementary material for: Adaptive DDoS detection mode in software-defined SIP-VoIP using transfer learning with boosted meta-learner
Source: PLoS One. 2025 Jun 26;20(6):e0326571. doi: 10.1371/journal.pone.0326571 (PMC12200684; doi:10.1371/journal.pone.0326571)
Supplement: Attachment — Submitted filename: Responses to Reviewers Comments R1.docx [file pone.0326571.s003.docx]

Review of the Paper: "Adaptive DDoS Detection Mode in Software-Defined SIP-VoIP Using Transfer Learning with Boosted Meta-Learner"

The paper proposes a transfer learning-based DDoS detection model for SIP-VoIP using BiGRU, BiLSTM, Random Forest, and XGBoost. While the topic is relevant and the approach promising, several areas require major revision to enhance clarity, justification, and practical applicability.

Key Revisions Needed:

- Classifier Justification: Explain why BiGRU, BiLSTM, and Random Forest were chosen over other models. Compare with alternative deep learning classifiers. Comments: As address in Section 1.2
- Dataset & Preprocessing: Provide details on feature selection, normalization, and handling of class imbalance. Clarify preprocessing steps. Comments: Pre-processing steps are detailed with the formulae for computation as in step-3 of Section 2 ‘Materials and Methods’. Data Balancing scheme as executed in Step-3 is detailed as suggested. Normalization is achieved in Step-5 using the standard normalizer of Equation 1.
- Related Work: Expand comparison with existing SIP-VoIP DDoS detection models and highlight novelty. Comments: As address in Section 1.2.
- Results & Explainability: Discuss false positives, false negatives, and model interpretability. Include confusion matrices, ROC curves, or SHAP analysis to strengthen insights. Comments: Model interpretability addressed in Section 3.1.
- Abstract & Conclusion: Add specific performance metrics and emphasize practical implications. Ensure clear takeaways. Comments: Revised Abstract
- Practical Deployment: Address real-time implementation, IDS integration, and robustness against adversarial attacks. Comments: See the Conclusion section for the implementation of the ensemble method as an embedded system application programming interface (API)
- Grammar & Readability: Improve structure, clarify research gaps, and refine writing for better comprehension.
