# Supplementary material for: Adaptive DDoS detection mode in software-defined SIP-VoIP using transfer learning with boosted meta-learner
Source: PLoS One. 2025 Jun 26;20(6):e0326571. doi: 10.1371/journal.pone.0326571 (PMC12200684; doi:10.1371/journal.pone.0326571)
Supplement: Attachment — Submitted filename: Responses to Reviewers Comments R2.docx [file pone.0326571.s004.docx]

Reviewer #1: - You need to provide a more thorough justification for choosing BiGRU, BiLSTM, and Random Forest over other potential models. Did you compare their performance with alternative deep learning classifiers? Including comparative analysis or reference results can strengthen your argument for your chosen classifiers. Comments: The utilization of Machine Learning (ML) schemes as data-driven model aids researchers to glean insightful knowledge for a domain task. However, a major issue with such models includes: (a) the more complex the model – the better its improved generalization and performance especially in handling large complex dataset, and (b) many of these traditional ML schemes such as Random Forest, SVM, AdaBoost, LR, GA, etc – have been known not to effectively handle categorical data. With these reasons as in Section 1.2 – researchers exploit deep learning (DL) scheme such as RNN, LSTM, BiLSTM, BiGRU, etc, as methods to address these inherent challenges with traditional ML.

- While you reference where preprocessing details can be found, you should explicitly state your feature selection criteria, normalization techniques, and methods for handling class imbalance directly in the main text. Make it easy for readers to understand each step without having to refer to other sections extensively. Comments: Addressed in Section 2 – Materials and Methods (with step-2) as marked ***red***.

- Expand upon the existing literature in the field of SIP-VoIP DDoS detection. Current citations seem limited. To clarify your contribution and novelty, you should discuss how your approach differs from or improves upon previous work. Comments: Literature Review addressed in Section 1.2 – while, contributions and novelty addressed in Section 1.3. It details how this study improves the existing works via its contributions as marked ***red***.

- A deeper analysis of your results is required. Discuss the implications of any false positives and false negatives you encountered and how they affect the model's reliability. Including visual aids such as confusion matrices and ROC curves is essential here, along with a discussion on model interpretability and potential avenues for improvement. Comments: Addressed in Section 3.1.

- In the Abstract, be sure to include specific performance metrics such as accuracy, precision, recall, and F1-score of your models. In your conclusion, highlight the practical implications of your work and how it can be applied in real-world scenarios, ensuring clear takeaways for the reader. Comments: Addressed in *Abstract*.

- Address how your DDoS detection model can be implemented in real-time and integrated into existing IDS frameworks. Discuss its robustness against adversarial attacks more comprehensively. This section should convince readers of the feasibility of your approach in a practical setting. Comments: Addressed in Section 3.1 Result and Findings.

- Focus on improving the overall structure and flow of the writing. Ensure technical jargon and terminology are clearly defined upon first use and maintain consistency throughout. This will aid comprehension for readers who may not be familiar with subject matter. Comments: Addressed.

- If you have visual data representation (e.g., graphs or tables), ensure they are properly labeled and referenced in the text. Each figure/table should have a clear legend or caption explaining what it represents. Comments: Addressed.

Reviewer #2: The manuscript is under journal scope but need to some modifications should be addressed as follows:

1. Provide a detailed justification for selecting these classifiers, including a comparison with alternative deep learning or machine learning models. Highlight their strengths and relevance to the problem being addressed. Comments: Addressed in Section 1.3

2. Describe the dataset in detail, including its size, source, and characteristics.
3. Include confusion matrices, ROC curves, precision-recall curves, or SHAP (SHapley Additive exPlanations) analysis to provide deeper insights into model performance. Comments: Addressed in Results and Findings Section 3.1.

4. Update the abstract to include key performance metrics (e.g., accuracy, F1-score, AUC-ROC). Comments: Addressed.

5. Discuss the robustness of the system against adversarial attacks and propose strategies to enhance security. Comments: Addressed in Discussion of Findings under the Section 3.1

6. Refine the language to ensure clarity and readability. Comments: Addressed.
